# Retinoid X receptor γ regulates epithelial–mesenchymal transition and tumor immune infiltration in papillary thyroid cancer tumorigenesis: an experimental and *in silico* study

**DOI:** 10.1530/EC-25-0015

**Published:** 2025-06-17

**Authors:** Pihong Li, Wei Zhang, Qiaolin Wu, Xiaohua Zhang, Zhouci Zheng

**Affiliations:** ^1^ Department of Neck Surgery, The Second Affiliated Hospital and Yuying Children’s Hospital of Wenzhou Medical University, Wenzhou, Zhejiang, China; ^2^ Departments of Breast Surgery, The First Affiliated Hospital of Wenzhou Medical University, Wenzhou, Zhejiang, China; ^3^ Departments of Anesthesiology, The First Affiliated Hospital of Wenzhou Medical University, Wenzhou, Zhejiang, China

**Keywords:** RXRG, papillary thyroid cancer, epithelial–mesenchymal transition, immune infiltration, lymph node metastasis

## Abstract

**Objective:**

This study aimed to elucidate the functional role and underlying molecular mechanisms of retinoid X receptor γ (RXRG) in the pathogenesis of papillary thyroid carcinoma (PTC).

**Methods:**

We analyzed RNA-seq data from The Cancer Genome Atlas database, ONCOMINE database, and Human Protein Atlas. RXRG expression was validated in 47 matched PTC-normal tissue pairs using real-time reverse transcription-polymerase chain reaction. Functional characterization was performed through loss- and gain-of-function experiments, complemented by flow cytometry analysis. Bioinformatics approaches were employed to investigate RXRG’s role in tumor immune infiltration.

**Results:**

RXRG was significantly upregulated in PTC (*P* < 0.001). Elevated RXRG expression correlated with aggressive clinicopathological features, including lymph node metastasis (*P* = 0.041), advanced tumor stage (*P* = 0.035), BRAF^V600E^ mutation (*P* < 0.001), and increase in tumor size (*P* = 0.011). Functional assays revealed that RXRG knockdown suppressed cell proliferation, colony formation, and migration capacity, whereas its overexpression promoted these oncogenic phenotypes. Mechanistically, RXRG regulated epithelial–mesenchymal transition (EMT) through modulation of E-cadherin, N-cadherin, vimentin, and key transcription factors (Snail and Slug). Furthermore, RXRG expression considerably influenced tumor immune infiltration patterns, particularly affecting eosinophils, NK cells, and B cells.

**Conclusion:**

Our study identifies RXRG as a novel oncogenic driver in PTC that promotes tumor progression through EMT regulation and immune microenvironment modulation.

## Introduction

Thyroid cancer is one of the most prevalent malignancy of the endocrine system and has been rapidly increasing in incidence worldwide (1). The incidence of thyroid cancer has been increasing globally by approximately 4% annually (2). In the United States, 43,800 newly diagnosed cases and 2,230 deaths were reported in 2022 (3). Meanwhile, in China, thyroid cancer ranks ninth among men and fourth among women, accounting for 38% of all new cases globally (4).

Papillary thyroid carcinoma (PTC) constitutes 80–85% of all thyroid malignancies, characterized by frequent lymph node metastasis (LNM) despite generally favorable prognosis (5). Genomic variations, including the activation of oncogenes and the inactivation of tumor suppressor genes, play a crucial role in the initiation and progression of thyroid cancer (6, 7, 8, 9, 10). Hence, a comprehensive understanding of the underlying pathogenesis and etiology of PTC is crucial for the identification of effective treatments and potential diagnostic biomarkers.

Retinoids, through their interactions with retinoic acid receptors (RARs) and retinoid X receptors (RXRs), play a critical role in regulating gene expression and controlling cell growth and differentiation (11, 12). The abnormal expression of retinoid X receptor γ (RXRG) is associated with certain diseases (13, 14, 15, 16, 17), but its role in the tumorigenesis and development of thyroid tumors remains unclear.

RXRG dysregulation may contribute to tumor dedifferentiation and metastatic progression in thyroid cancer, warranting systematic investigation. Thus, we conducted a large-scale comparison of RXRG expression levels between PTC tumors and normal tissues. In addition, we analyzed the relationship between RXRG expression and clinical features and examined the function of RXRG in PTC cell lines. Bioinformatics analysis was performed to explore the correlation between RXRG expression and tumor immune microenvironment. Overall, the aim of this study was to investigate the role of RXRG in PTC.

## Materials and methods

### ONCOMINE database

By meta-analysis of the Oncomine database (18), we analyzed RXRG gene expression levels between normal and PTC tissues in three distinct thyroid cancer datasets. Transcriptional expression data of RXRG were downloaded, and a Student's t-test was performed to assess the differences in expression. The cutoff values for statistical significance were set at a *P*-value of 0.01 and a fold change of 1.5, data type: mRNA.

### Human Protein Atlas

In this study, we utilized immunohistochemistry images from the Human Protein Atlas (19), including 495 primary PTC tumors and 653 normal thyroid tissues, to compare the protein expression of RXRG between normal and primary PTC tissues.

### The Cancer Genome Atlas (TCGA) database

The clinical data and corresponding RNA-seq data of RXRG, including 465 primary PTC tumors and 59 normal thyroid tissues, were downloaded from the TCGA database (20).

### Patients and samples

We collected 47 paired primary PTC cancerous tissues and their matched non-cancerous thyroid tissues from patients who underwent initial surgery. These samples were histologically confirmed and retrospectively classified by two senior pathologists. Written informed consent was obtained from each patient for the publication of clinical data. This study complies with all regulations.

### RNA extraction and real-time reverse transcription-polymerase chain reaction

Total RNA was extracted from the samples using TRIzol reagent (Life Technologies, USA), following the manufacturer’s instructions. The cDNA preparation was performed using the ReverTra Ace® qPCR RT Kit (Toyobo, Japan). Real-time reverse transcription-polymerase chain reaction (RT-qPCR) was carried out using the Thunderbird SYBR qPCR Mix (Toyobo, Japan). The cycle threshold (Ct) values were analyzed using the comparative Ct (2^−^^ΔΔCt^) method. The primer sequences for RT-qPCR are listed in Supplementary Table 2 (see section on Supplementary materials given at the end of the article). Each sample was run in triplicate, and the internal control used was GAPDH.

### Protein extraction and western blot analysis

For protein extraction, the indicated cells were lysed in RIPA lysis buffer (Beyotime, China). Equal amounts of proteins were separated by sodium dodecyl sulfate polyacrylamide gel electrophoresis (SDS-PAGE) and transferred onto a polyvinylidene fluoride (PVDF) membrane. β-actin protein was used as a control. Antibodies against N-cadherin, E-cadherin, β-catenin, vimentin, Akt, pAkt, and β-actin were purchased from Cell Signaling Technology (USA).

### Cell lines and cell culture

The human thyroid cancer cell lines TPC1, BCPAP, and KTC were authenticated. All cell lines were cultured at 37°C with 5% CO_2_ in RPMI 1640 medium supplemented with 10% fetal bovine serum (Life Technologies, USA), 1× MEM nonessential amino acids, and 1× sodium pyruvate.

### RNA interference and lentiviral infections

To knock down the expression of RXRG, small interfering RNA (siRNA) specifically targeting RXRG and control siRNA (siNC) were obtained from Gene Pharma (China). Cell transfection was performed using Lipo iMAX (Invitrogen, USA), with a final siRNA concentration of 100 nM for TPC1 cells and 50 nM for BCPAP and KTC cells. After 48 h of transfection, cells were collected for the analysis of RXRG expression. The sequences of siRNA are as follows: RXRG, sense 5′-CUA​CAC​GUG​UCG​GGA​UAA​UTT-3′, antisense 5′-AUU​AUC​CCG​ACA​CGU​GUA​GTT-3′. All experiments were repeated three times.

Lentiviral particles containing RXRG miRNA were purchased from GeneChem (China). TPC1 cells were transfected with lentiviral particles carrying NC/RXRG (lenti-NC/lenti-RXRG), according to the manufacturer’s instructions. The expression level of RXRG was assessed by RT-qPCR 1 week after screening.

### Cell proliferation and colony formation assay

Cell proliferation was evaluated using the CCK8 assay. The cells of interest were seeded and cultured in 96-well plates. At predetermined time points, 10 μL CCK8 solution (Beyotime, China) was added to the medium, and the plates were incubated for an additional 2 h. The absorbance of the plates was measured using a microplate reader at a wavelength of 450 nm. All CCK8 assays were repeated three times.

For the colony formation assay, monolayer culture was employed. The cells were seeded into 6-well plates, and the medium was refreshed every 48 h. After 8–14 days of culture, the surviving colonies were fixed with 4% paraformaldehyde (PFA, Sigma, USA). Subsequently, the colonies were stained with 0.01% crystal violet and the number of colonies was counted. All colony formation assays were repeated three times.

### Transwell migration assay

To assess cell migration, we employed transwell cell culture chambers (Corning Costar Corp, USA). The cells of interest were seeded onto the top chamber in medium containing 10% FBS, while the bottom chamber was filled with the same concentration of medium. After 24 h of seeding, the bottom membranes were fixed with 4% paraformaldehyde (PFA). Subsequently, the membranes were stained with 0.01% crystal violet and photographed under a microscope at a magnification of 200×.

### Flow cytometry analysis

For cell apoptosis assays, we utilized the Annexin V-APC/7-AAD apoptosis kit (MultiSciences Biotech, China). The cells of interest were seeded and cultured in 6-well plates. At specific time points, the cells were harvested and washed with PBS. Then, the cells were stained with V-ACP dye and 7-AAD dye for 15 min. Flow cytometry analysis was performed using the FACSCalibur instrument (Becton Dickinson, USA), and the data were analyzed using the FlowJo7.6.1 software.

### Statistical analysis

The expression difference between PTC tumors and adjacent normal tissues in the validated cohort and the TCGA cohort was evaluated using the Mann–Whitney U test. The relationship between clinicopathological characteristics and RXRG expression was assessed using the chi-square test or Fisher’s exact test. Logistic regression analysis was performed to assess the lymph node metastatic risk of RXRG. The single-sample sequence set enrichment analysis (ssGSEA) from the R package was used to evaluate the correlation between RXRG expression and immune cells. All *P*-values were two-sided, and a *P*-value of less than 0.05 was considered statistically significant. The statistical analysis was conducted using the SPSS version 18.0 (USA). Graphs were generated using the GraphPad Prism 5 (GraphPad Software, USA) or ggplot 2 (R studio. 3.3.6).

## Results

### RXRG was overexpressed in PTC

To evaluate the clinical significance of RXRG in PTC, we systematically analyzed its expression patterns at transcriptional and translational levels. We incorporated data from the ONCOMINE database and Human Protein Atlas into our multiplatform investigation to assess RXRG prognostic and therapeutic potential. As shown in Supplementary Figure 1A, considerable increased mRNA expression of RXRG was found in head and neck cancer. In the Vasko dataset (21), PTC tissues showed RXRG overexpression compared with normal tissues with a fold change of 8.643 (*P* = 1.25E-10). Meanwhile, Giordano (22) observed 1.709 fold change in RXRG expression in PTC samples (*P* = 6.92E-9) and He (23) found 8.096 fold increase in RXRG mRNA expression in PTC tissues (*P* = 6.64E-6), as shown in Supplementary Table 1. At the protein level, immunohistochemical analysis showed detectable RXRG expression in the PTC specimens, whereas no expression was observed in the normal thyroid tissues (Supplementary Figure 1B).

**Figure 1:**
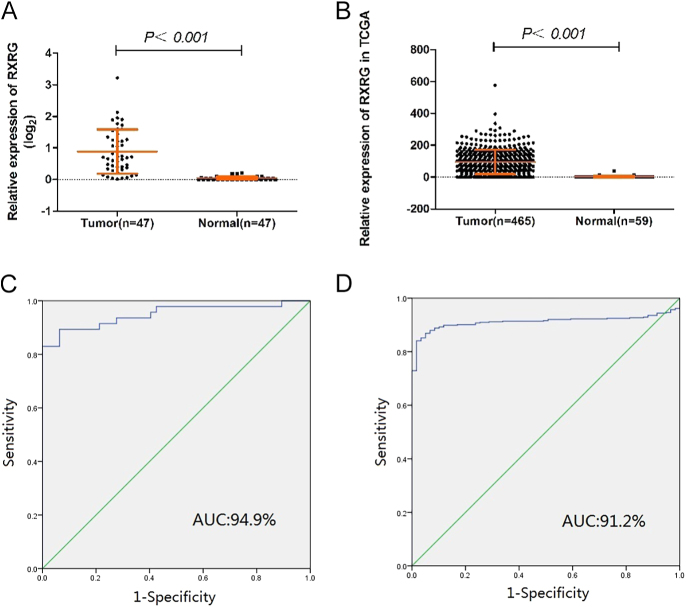
RXRG expression was significantly upregulated in thyroid cancer samples compared with normal thyroid tissues in validated and TCGA cohorts. (A) In the validated cohort, RXRG expression was examined by qRT-PCR in 47 paired PTC samples and adjacent normal tissues (Mann–Whitney U-test, *P* < 0.001). (B) The TCGA cohort contained 465 tumor samples and 59 normal tissue samples. (C) ROC curve for RXRG expression to diagnose PTC in the validated cohort. (D) ROC curve for RXRG expression to diagnose PTC in the TCGA cohort.

Then, the mRNA expression of RXRG in 47 pairs of PTC tumor tissues and matched adjacent noncancerous tissues was detected by RT-qPCR to validate the aforementioned results. As shown in Fig. 1A, the expression of RXRG in the PTC tumor samples was significantly overexpressed compared with that in the matched normal tissues in the validated cohort (T: *n* = 0.88 ± 0.70: 0.05 ± 0.047, *P* < 0.001). This observation was corroborated by TCGA data analysis, which showed markedly higher RXRG expression in PTC samples (94.86 ± 76.34) versus normal thyroid tissue (2.45 ± 5.57; *P* < 0.001, Fig. 1B). Moreover, we plotted a receiver operator characteristic (ROC) curve to determine the diagnostic value of RXRG. In the validated cohort, the area under the ROC curve (AUC) was 94.9% (95% CI: 90.2–99.6%), with 89.4% sensitivity and 93.6% specificity. Moreover, sensitivity and specificity rates of 89.9 and 88.1% were achieved in the TCGA cohort (AUC: 91.2%, 95% CI: 88.7–93.7%) in discriminating PTC tumors from normal thyroid tissues (Fig. 1C and D). These robust metrics establish RXRG as a highly specific molecular marker capable of distinguishing malignant from normal thyroid tissue.

### Relationship between RXRG expression and clinicopathological features

To elucidate the clinical relevance of RXRG in PTC pathogenesis, we systematically evaluated its correlation with key clinicopathological parameters across our validation cohort and the TCGA dataset. In the validated cohort, the results demonstrated a nonsignificant trend (*P* > 0.05) toward association between elevated RXRG expression and advanced disease features, including LNM and higher clinical stage (Table 1). However, analysis of the larger TCGA cohort revealed statistically significant correlations between high RXRG expression and several aggressive clinicopathological features: increase in tumor size (*P* = 0.011), presence of LNM (*P* = 0.041), advanced clinical stage (*P* = 0.035), specific histological subtypes (*P* = 0.008), and BRAF^V600E^ mutation status (*P* < 0.001) (Table 1). Notably, RXRG expression showed no association with age or gender in either cohort (*P* > 0.05).

**Table 1:** The relationship between RXRG expression and clinicopathological features in the validated cohort and TCGA cohort.

Clinicopathological features	The validated cohort (*n* = 47)			The TCGA cohort (*n* = 465)		
Age (year)	Low expression (*n* = 15) (%)	High expression (*n* = 32) (%)	*P* value	Low expression (*n* = 232) (%)	High expression (*n* = 233) (%)	*P* value
Mean	50.46 ± 10.65	53.56 ± 13.14	0.43	47.76 ± 16.23	46.88 ± 15.29	0.651
>45	12 (80.0)	20 (62.5)	0.23	123 (53.0)	123 (52.8)	0.961
≤45	3 (20.0)	12 (37.5)		109 (47.0)	110 (47.2)	
Gender			0.697			0.977
Male	1 (6.7)	5 (15.6)		63 (27.2)	63 (27.0)	
Female	14 (93.3)	27 (84.4)		169 (72.8)	170 (73.0)	
Tumor size (mm)						
Mean	16.60 ± 9.74	15.91 ± 10.60	0.639	28.63 ± 18.03	25.30 ± 17.21	0.011
Tumor stage			0.328			0.64
T1	6 (40.0)	9 (28.1)		63 (27.2)	71 (30.5)	
T2	4 (26.7)	8 (25.0)		81 (34.9)	70 (30.0)	
T3	4 (26.7)	11 (34.4)		76 (32.8)	82 (35.3)	
T4	1 (6.7)	4 (12.5)		12 (5.2)	10 (4.3)	
LNM			0.083			0.041
Yes	8 (53.3)	25 (78.1)		89 (38.4)	123 (52.8)	
No	7 (46.7)	7 (21.9)		114 (49.1)	106 (45.5)	
BRAF^V600E^ mutation						<0.001
Yes				78 (33.6)	136 (58.3)	
No				149 (64.2)	90 (38.6)	
Histological type						0.008
Classical				150 (64.7)	177 (76.0)	
Others				82 (35.3)	56 (24.0)	
Clinical stage			0.13			0.035
I	4 (26.7)	9 (28.1)		137 (59.1)	124 (53.2)	
II	2 (13.3)	0 (0.0)		30 (12.9)	17 (7.3)	
III	7 (46.7)	14 (43.8)		44 (19.0)	61 (26.2)	
IV	2 (13.3)	9 (28.1)		21 (9.1)	30 (12.9)	

LNM, lymph nodes metastasis.

The relationship between RXRG expression and LNM was then further analyzed. Univariate logistic regression analysis showed that the significant variables for LNM were age (OR 0.618, 95% CI: 0.422–0.904, *P* = 0.013), histological type (OR 0.41, 95% CI: 0.263–0.640, *P* < 0.001), tumor stage, and RXRG expression (OR 1.486, 95% CI: 1.017–2.173, *P* = 0.041) (Table 2). Meanwhile, gender and BRAF^V600E^ mutation status showed suggestive trends, but the associations did not reach statistical significance (*P* > 0.05). Multivariate analysis incorporating these variables confirmed RXRG expression as an independent predictor of LNM (OR 1.598, 95% CI 1.043–2.448; *P* = 0.031), along with tumor stage. Conversely, increasing age (OR 0.509, 95% CI 0.332–0.782; *P* = 0.02) and certain histological subtypes (OR 0.454, 95% CI 0.277–0.742; *P* < 0.001) emerged as protective factors against nodal metastasis (Table 3). In summary, RXRG was an independent risk indicator of LNM in patients with PTC.

**Table 2:** Univariate logistic regression analysis for the lymph node metastatic risk.

Clinicopathological features	OR	95% CI	*P* value
Age	0.618	0.422–0.904	0.013
Gender	1.526	0.998–2.332	0.051
Tumor stage			
T1	Reference	Reference	
T2	1.768	1.069–2.924	0.026
T3	3.125	1.901–5.138	<0.001
T4	8.707	2.753–27.538	<0.001
Histological type	0.41	0.263–0.640	<0.001
BRAF^V600E^ mutation	1.463	0.996–2.149	0.051
RXRG expression	1.486	1.017–2.173	0.041

**Table 3:** Multivariate logistic regression analysis for the lymph node metastatic risk.

Clinicopathological features	OR	95% CI	*P* value
Age	0.509	0.332–0.782	0.02
Gender	1.430	0.890–2.297	0.139
Tumor stage			
T1	Reference	Reference	
T2	1.642	0.965–2.795	0.067
T3	3.689	2.151–6.326	<0.001
T4	10.805	3.275–35.65	<0.001
Histological type	0.454	0.277–0.742	<0.001
BRAF^V600E^ mutation	1.225	0.789–1.881	0.354
RXRG expression	1.598	1.043–2.448	0.031

### The downregulation of RXRG inhibited PTC cell proliferation, colony formation, and migration

**Figure 2:**
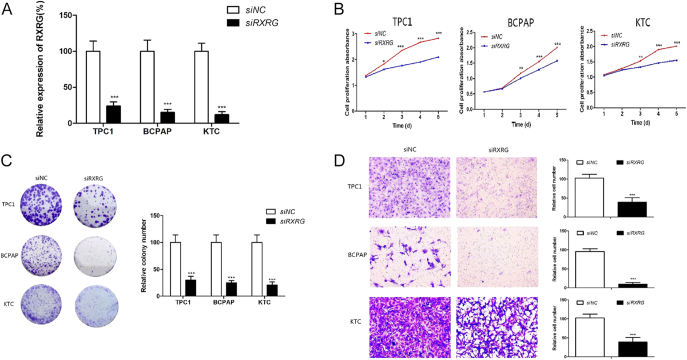
Downregulation of RXRG inhibited PTC cell proliferation, colony formation, and migration. (A) Knockdown of RXRG was confirmed by quantitative reverse transcription-polymerase chain reaction (RT-qPCR). (B) RXRG knockdown significantly inhibited cell proliferation. (C) RXRG knockdown significantly inhibited colony formation. (D) Knockdown of RXRG significantly impaired cell migration ability. *P < 0.05, **P < 0.01, ***P < 0.001.

Given that RXRG is frequently overexpressed in PTCs, this gene may play an oncogenic role in thyroid tumorigenesis. Thus, the expression of RXRG was downregulated by using targeted siRNA. RT-qPCR was performed to confirm the efficiency of RXRG knockdown by the targeted siRNA (Fig. 2A). The results revealed that RXRG knockdown inhibited thyroid cancer cell proliferation and colony formations compared with the control (Fig. 2B and C). Furthermore, transwell assay revealed that RXRG knockdown could lower the migration capacity of PTC cell lines. As shown in Fig. 2D, the cell lines transfected with siRXRG migrated significantly less cells than the control cells after 24 h of seeding (*P* < 0.001) Overall, the concordant suppression of proliferation, clonogenicity, and migration following RXRG knockdown strongly supports its role as an oncogene in thyroid tumorigenesis.

### The overexpression of RXRG promoted PTC cell proliferation, colony formation, and migration

**Figure 3:**
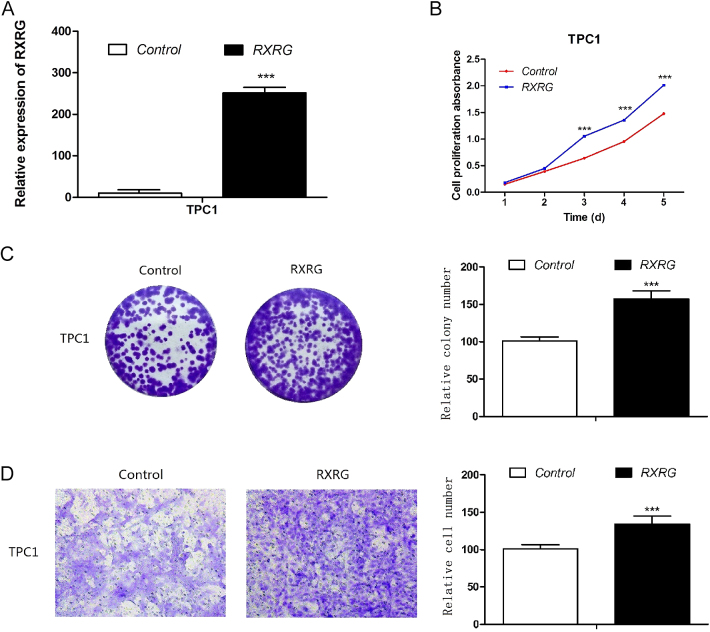
Overexpression of RXRG promoted PTC cell proliferation, colony formation, and migration. (A) Overexpression of RXRG was confirmed by quantitative reverse transcription-polymerase chain reaction (RT-qPCR). (B) Overexpression of RXRG significantly promoted cell proliferation. (C) Overexpression of RXRG significantly promoted colony formation. (D) Overexpression of RXRG significantly enhanced cell migration ability. *P < 0.05, **P < 0.01, ***P < 0.001.

To complement our loss-of-function studies, we selected the TPC1 cell line from our panel of PTC models to examine the consequences of RXRG overexpression. RT-qPCR analysis was conducted to confirm the successful overexpression of RXRG (Fig. 3A). The data showed that RXRG overexpression caused a significant increase in cell proliferation (Fig. 3B, *P* < 0.001). The stimulative effect of the overexpressed RXRG on cell growth was further proven by colony formation assay. The colonies formed in the RXRG-overexpression group increased compared with those in the control group (Fig. 3C, *P* < 0.001). Furthermore, RXRG overexpression promoted the cell migration capabilities of TPC1 compared with the control cells (Fig. 3D, *P* < 0.001). These gain-of-function experiments provide reciprocal validation of our knockdown results, demonstrating that RXRG not only maintains but actively drives malignant behavior in PTC cells.

### RXRG inhibits cell apoptosis in thyroid cancer cells

**Figure 4:**
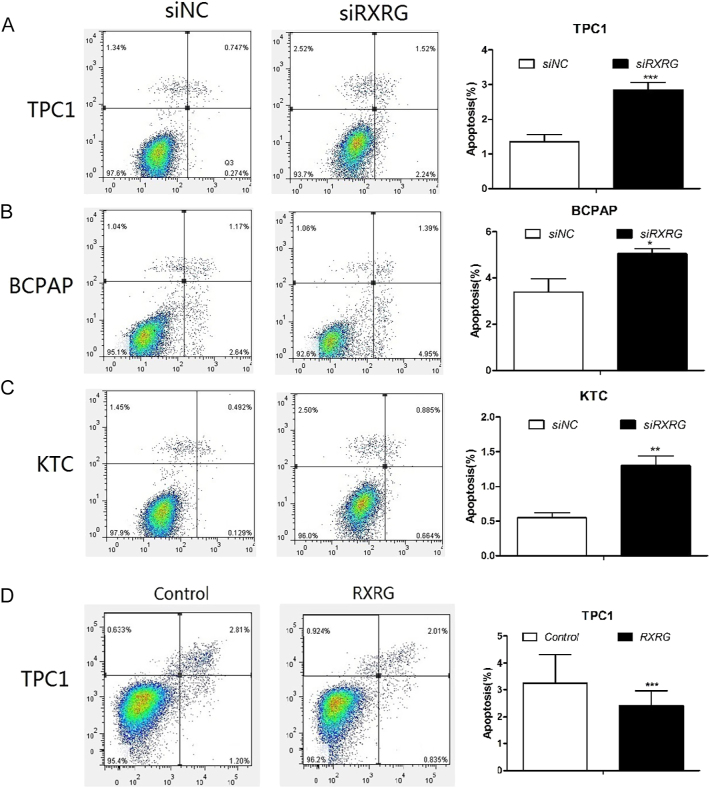
RXRG inhibits cell apoptosis in thyroid cancer cells. (A–C) RXRG knockdown significantly induced cell apoptosis as compared to the control in PTC cell lines. (A: TPC1, B: BCPAP, C: KTC). (D) Overexpression of RXRG leads to a decrease in cell apoptosis as compared to the control in TPC1 cells. *P < 0.05, **P < 0.01, ***P < 0.001.

To evaluate the role of RXRG in programmed cell death regulation, we systematically examined apoptosis rates following the regulation of RXRG expression across multiple PTC cell lines. RXRG knockdown induced cell apoptosis compared with that in the control. The percentage of apoptotic cells increased from 1.02 ± 0.12 to 3.67 ± 0.23 in the TPC1 cells (*P* < 0.001), from 3.75 ± 0.17 to 6.42 ± 0.33 in the BCPAP cells (*P* = 0.027), and from 0.62 ± 0.14 to 1.55 ± 0.27 in the KTC cells (*P* = 0.005; Fig. 4A, B, C). By contrast, the overexpression of RXRG in the TPC1 cells led to a decrease in cell apoptosis compared with that in the control (4.01 ± 0.87 vs 2.85 ± 0.43, *P* < 0.001, Fig. 4D). These findings indicated that RXRG functions as a potent suppressor of apoptosis in PTC cells, suggesting that its oncogenic activity is mediated, in part, by mechanisms that enhance cell survival.

### RXRG promotes cell metastasis by regulating the process of EMT and the expression of metastasis-related genes in thyroid cancer

**Figure 5:**
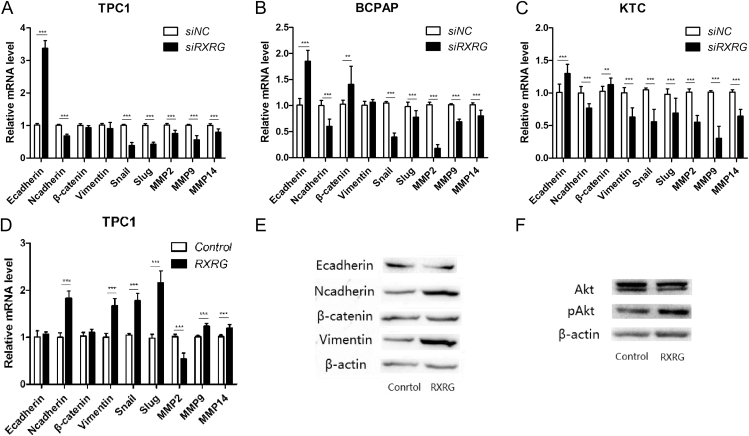
RXRG promotes cell metastasis by regulating the process of epithelial–mesenchymal transition (EMT) and the expression of metastasis-related genes in thyroid cancer. (A) The metastasis-related genes involved in the EMT process were evaluated by RT-qPCR in TPC1. (B) The metastasis-related genes involved in the EMT process were evaluated by RT-qPCR in BCPAP. (C) The metastasis-related genes involved in the EMT process were evaluated by RT-qPCR in KTC. (D) Ectopic expression of RXRG promoted the EMT process by regulating the expression of metastasis-related genes in TPC1 cells. (E) The metastasis-related genes involved in the EMT process were evaluated by western blot in TPC1. (F) RXRG overexpression activated the activities of the PI3K/Akt pathway by increasing the phosphorylation of Akt. *P < 0.05, **P < 0.01, ***P < 0.001.

To elucidate the mechanistic basis of RXRG-mediated metastasis, we investigated its role in EMT and associated signaling pathways. The results indicated that RXRG knockdown inhibited the expression of mesenchymal markers (N-cadherin and vimentin) and increased that of epithelial markers (E-cadherin and β-catenin) in at least one cell line (Fig. 5A, B, C). Conversely, the ectopic expression of RXRG in TPC1 cells extensively upregulated the expression of mesenchymal markers (N-cadherin and vimentin) and slightly decreased the expression of epithelial markers (E-cadherin; Fig. 5D). The results of Western blot analysis were consistent with the results of RT-qPCR. As shown in Fig. 5E, N-cadherin and vimentin considerably increased after RXRG overexpression. Snail and Slug are transcription factors known to play roles in EMT during tumorigenesis (24). The effect of RXRG on the expression of these transcription factors in thyroid cancer cell lines was investigated. RXRG knockdown considerably decreased the expression of Snail and Slug in the PTC cell lines (Fig. 5A, B, C), whereas the overexpression of RXRG in TPC1 cells could considerably increase their expression (Fig. 5D). The effect of altered expression of RXRG on the expression of matrix metalloproteinases (MMPs) in the PTC cell lines was examined. RXRG knockdown caused a downregulation in the transcription of MMPs in the PTC cell lines (Fig. 5A, B, C). By contrast, the overexpression of RXRG in the TPC1 cells efficiently increased MMP (MMP9 and MMP14) expression (Fig. 5D). In addition, we investigated the role of RXRG on the MAPK and PI3K/Akt pathways, which are important signaling pathways in tumorigenesis (8). RXRG overexpression activated the activities of the PI3K/Akt pathway by increasing the phosphorylation of Akt (Fig. 5F). These coordinated effects on EMT markers, transcriptional regulators, proteolytic enzymes, and survival pathways establish RXRG as a critical mediator of metastatic progression in PTC.

### RXRG regulates tumor immune infiltration in PTC

**Figure 6:**
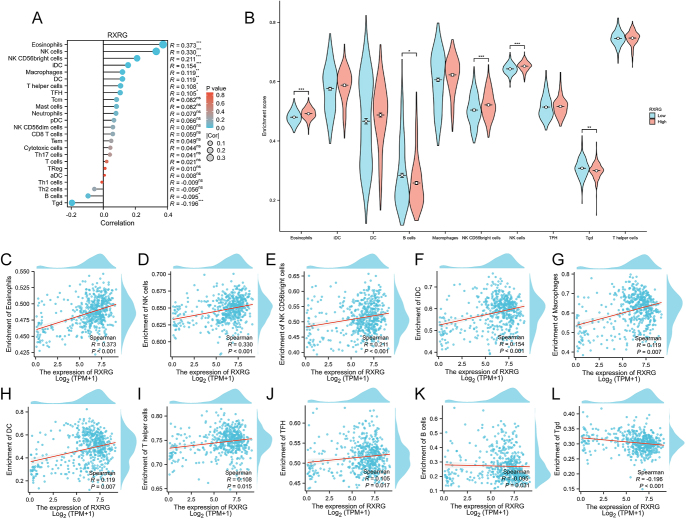
Relationship between RXRG expression and immune infiltration in PTC microenvironment. (A) Correlation between the relative abundance of 24 immune cells and RXRG expression. The size of the dots indicates the absolute value of Spearman’s correlation coefficient R. (B) Correlation between high and low RXRG expression and the infiltration levels of immune cells in PTC. (C–L) Correlation between RXRG expression and infiltration levels of eosinophils (C), NK cells (D), NK CD56^bright^ cells (E), iDC (F), macrophages (G), DC (H), T helper cells (I), TFH (J), B cells (K), and Tgd (L). *P < 0.05, **P < 0.01, and ***P < 0.001.

Comprehensive immune profiling revealed considerable alterations in tumor-infiltrating leukocyte populations associated with RXRG expression levels (Fig. 6A), including eosinophils, NK cells, NK CD56^bright^ cells, immature dendritic cells (iDC), macrophages, dendritic cells (DC), T helper cells, T follicular helper (TFH), B cells, and T gamma delta (Tgd) cells (Fig. 6C, D, E, F, G, H, I, J, K, L). Furthermore, the enrichment score for eosinophils, NK CD56^bright^ cells, and NK cells were considerably higher in the high RXRG expression group than in the low RXRG expression group, whereas the enrichment score for B cells and Tgd were significantly lower in the high RXRG expression group (Fig. 6B, *P* < 0.05). The coordinated changes across multiple immune lineages suggest that RXRG influences more immune signaling networks than individual cell types. This immunomodulatory activity, combined with RXRG’s direct oncogenic effects on tumor cells, likely contributes to its strong association with aggressive clinical features in PTC.

## Discussion

The incidence of thyroid cancer, particularly PTC, has been rapidly increasing in recent years (2, 3). PTC has a relatively better prognosis than the other types of thyroid cancer, and the risk of LNM in patients with PTC can lead to locoregional recurrence and the need for additional surgeries (25, 26, 27). Despite remarkable advances in high-throughput genomic technologies that have identified numerous molecular alterations in PTC (28, 29), the fundamental mechanisms driving tumor initiation and metastatic progression remain incompletely characterized.

The dysregulation of RARs and RXRs, particularly the imbalance in the nuclear-to-cytoplasmic ratio of these proteins, plays an important role in the development and progression of thyroid cancer. Hoftijzer *et al.* found increased expression of cytoplasmic (c) RARA, cRARG, and cRXRB, and decreased expression of nuclear (n) RARB, nRARG, and nRXRA in thyroid carcinomas compared with benign tissues (30). In addition, Takiyama *et al.* revealed that the nuclear expression of RXRs (α, β, and γ) was lower in thyroid carcinomas than in normal tissues (31). The differential expression of RARs and RXRs in thyroid carcinomas might be implicated in the pathogenesis of thyroid cancer.

Previous studies have suggested that abnormal expression of the RXRG gene is implicated in various diseases. Lee *et al.* found that the methylation of RXRG has been identified as a prognostic factor in non-small cell lung cancer (14). Moreover, Kalra *et al.* reported that the downregulation of RXRG has been associated with increased resistance to apoptosis in epithelial ovarian cancer (13). In addition, RXRG has been proposed as an independent prognostic biomarker in estrogen receptor-positive invasive breast cancer (16). In the context of thyroid cancer, the upregulation of RXRG has been linked to tumor dedifferentiation and LNM (17). However, the role of RXRG in PTC tumorigenesis remains unclear.

In our study, we investigated the expression of RXRG in PTC tumors and adjacent noncancerous tissues in a cohort of 47 patients, and the results validated a considerable increase in RXRG expression in PTC tumors compared with noncancerous tissues. This finding was consistent with the analysis of large-scale samples from the TCGA database, ONCOMINE database, and Human Protein Atlas. Furthermore, clinicopathological analysis revealed a positive association between RXRG expression and LNM. Based on these findings, we propose that PTC patients with elevated RXRG expression indicate an increase in prognostic risk. Therefore, we recommend more comprehensive surgical intervention for these patients, including (but not limited to) central regional lymph node dissection and lateral neck lymph node dissection. During postoperative follow-up, quarterly neck ultrasound examinations are recommended for the first 2 years after surgery. Particular attention should be devoted to cervical lymph node status in this patient subgroup to facilitate the early detection of potential recurrence. In addition to promoting cell metastasis through the induction of EMT, RXRG facilitated thyroid tumorigenesis by enhancing cell proliferation, colony formation, and resistance to cell apoptosis.

Numerous studies have demonstrated that cadherin switching plays a crucial role in regulating the migratory and invasive abilities of cells during EMT (32, 33, 34). Our data showed a notable increase in E-cadherin and a decrease in N-cadherin upon the knockdown of RXRG in the PTC cell lines. Conversely, the overexpression of RXRG considerably increased the expression of N-cadherin in the TPC1 cells. Furthermore, our data revealed that RXRG depletion considerably inhibited the expression of EMT regulatory factors, such as Snail and Slug, in the PTC cell lines. Conversely, the ectopic expression of RXRG upregulated the expression of these regulatory factors. These findings suggest that RXRG enhances the migratory capabilities of PTC cells by transcriptionally regulating EMT-related proteins, including E-cadherin, N-cadherin, Snail, and Slug. In addition, we investigated the effect of RXRG on the transcriptional regulation of MMPs in PTC cells. Our results indicated that the downregulation of RXRG greatly suppressed the expression of MMP-2, MMP-9, and MMP-14, whereas the overexpression of RXRG in the TPC1 cells increased the expression of MMP-9 and MMP-14. These results provide a mechanistic basis for the observed clinical association between RXRG overexpression and LNM, suggesting RXRG is a potential therapeutic target in advanced PTC.

The alteration of the tumor microenvironment considerably influences tumorigenesis, and the role of immune cells in tumor progression varies across different contexts. Tumor-associated tissue eosinophilia (TATE) is associated with improved prognosis in various solid malignancies (35, 36, 37). However, in patients with Hodgkin’s lymphoma, TATE may be linked to an increased risk of poor outcomes (38). In addition, elevated levels of NK cells can improve the innate immune response against oncogenesis, leading to the favorable prognoses of patients with cancers (39). The localization of B cells within secondary lymphoid organs has been correlated with effective antitumor control (40). In the present study, we found that high RXRG expression was positively correlated with eosinophils, NK CD56^bright^ cells, and NK cells, and negatively associated with B cells and Tgd. These immunomodulatory effects, combined with RXRG’s direct oncogenic activities, likely contribute to its association with the aggressive clinical features in PTC.

This study has several limitations. First, the absence of data on disease-free survival constrains the evaluation of the prognostic value of RXRG in PTC. Future studies should include comprehensive data collection to further investigate the clinical implications of RXRG expression in patients with PTC. Second, the results of bioinformatics analyses presented in this study are primarily based on theoretical data and should be validated through *in vivo* and *in vitro* experiments. A RXRG expression was linked to BRAF^V600E^ mutation, a major activator of the MAPK pathway; we did not investigate its impact on MAPK signaling. Future studies are needed to explore this potential connection.

In conclusion, our findings suggest that RXRG is commonly overexpressed in primary PTC and exerts oncogenic functions in PTC tumorigenesis. Our data indicated that RXRG regulates tumor immune infiltration and promotes thyroid tumorigenesis by enhancing cell proliferation, colony formation, metastasis, and suppressing cell apoptosis. Furthermore, RXRG may enhance the migratory capacity of PTC cells through EMT. However, further research is required to fully elucidate the role of RXRG in PTC and its underlying mechanisms.

## Supplementary materials



## Declaration of interest

The authors declare that there is no conflict of interest that could be perceived as prejudicing the impartiality of the work reported.

## Funding

This work was supported by the Wenzhou Science and Technology Project (Y20210176) and the Natural Science Foundation of Zhejiang Province (LTGY23H160020).

## Ethical approval

Ethical approval for this study was obtained from the Ethical Committee of the Second Affiliated Hospitals of Wenzhou Medical University (LCKY2020-38).
