# Supplementary material for: Retinoid X receptor γ regulates epithelial–mesenchymal transition and tumor immune infiltration in papillary thyroid cancer tumorigenesis: an experimental and in silico study
Source: Endocr Connect. 2025 Jun 17;14(6):e250015. doi: 10.1530/EC-25-0015 (PMC12177880; doi:10.1530/EC-25-0015)
Supplement: Supplementary file 1 [file supplementary_materials.pdf]

Supplementary Figure 1. RXRG was overexpressed in PTC.

Note: (A) Transcriptional expression of RXRG in 20 different types of cancer diseases (ONCOMINE database). (B) Representative immunohistochemistry images of RXRG in PTC tissue and normal thyroid tissue (Human Protein Atlas).

Supplementary Table 1. Significant changes of RXRG expression in transcription level between PTC and normal thyroid tissues (ONCOMINE database).

Database	Type	Fold Change	P value	t-test
Vasko Thyroid	PTC	8.643	1.25E-10	14.970
Giordano Thyroid	PTC	1.709	6.92E-9	12.532
He Thyroid	PTC	8.096	6.64E-6	9.183

Supplementary Table 2. The primer sequences for RT-qPCR

Gene	Forward (5’-3’)	Reverse (5’-3’)
GAPDH	GGTCGGAGTCAACGGATTTG	ATGAGCCCCAGCCTTCTCCAT
RXRG	GCCCCGGATCTCTGGTTAAA	TTGACTACATGGGCCTGCAT
Ecadherin	CGAGAGCTACACGTTCACGG	GGGTGTCGAGGGAAAAATAGG
Ncadherin	AGCCAACCTTAACTGAGGAGT	GGCAAGTTGATTGGAGGGATG
β-catenin	CTCTTCCAGCCTTCCTTCCT	AGCACTGTGTTGGCGTACAG
Vimentin	CCTCACCTGTGAAGTGGATGC	CAACGGCAAAGTTCTCTTCCA
Snail	CCTTCTCTAGGCCCTGGCT	AGGTTGGAGCGGTCAGC
Slug	TCGGACCCACACATTACCTT	GCAGTGAGGGCAAGAAAAAG
MMP2	GGAAAGCCAGGATCCATTTT	ATGCCGCCTTTAACTGGAG
MMP9	ACGACGTCTTCCAGTACCGA	GCACTGCAGGATGTCATAGG
MMP14	GTGGTCTCGGACCATGTCTC	GGTAGCCATATTGCTGTAGCC
